# Different Types of Periampullary Duodenal Diverticula Are Associated with Occurrence and Recurrence of Bile Duct Stones: A Case-Control Study from a Chinese Center

**DOI:** 10.1155/2016/9381759

**Published:** 2016-04-06

**Authors:** Zhen Sun, Wenhui Bo, Ping Jiang, Quan Sun

**Affiliations:** Department of General Surgery, Zhongnan Hospital of Wuhan University, No. 169 Donghu Road, Wuchang District, Wuhan 430071, China

## Abstract

*Aims*. We here investigated the association of different types of periampullary diverticula (PAD) with pancreaticobiliary disease and with technical success of endoscopic retrograde cholangiopancreatography (ERCP).* Methods*. A total of 850 consecutive patients who underwent their first ERCP were entered into a database. Of these patients, 161 patients (18.9%) had PAD and the age- and sex-matched control group comprised 483 patients.* Results*. PAD was correlated with common bile duct (CBD) stones (59.6% versus 35.0% in controls; *P* = 0.008) and negatively correlated with periampullary malignancy (6.8% versus 21.5% in controls; *P* = 0.004). The acute pancreatitis was more frequent (62.5%) in patients with PAD type 1 followed by PAD type 2 (28.9%, *P* = 0.017) and type 3 (28.0%, *P* = 0.006). No significant differences were observed in successful cannulation rate and post-ERCP complications among the 3 types of PAD. Type 1 PAD patients had less recurrence of CBD stones than did the patients who had type 2 or type 3 PAD (53.8% versus 85.7%; *P* = 0.043).* Conclusions*. PAD, especially type 1 PAD, is associated with an increased acute pancreatitis as well as occurrence and recurrence of CBD stones. PAD during an ERCP should not be considered as an obstacle to a successful cannulation.

## 1. Introduction

Periampullary diverticula (PAD), also known as perivaterian or peripapillary diverticula, is extraluminal mucosal outpouching of the duodenal arising within a radius of 2-3 cm from the ampulla of Vater [1]. PAD are observed in around 10–20% of patients undergoing endoscopic retrograde cholangiopancreatography (ERCP) [2] and their incidence increases with age. There are three types of PAD according to the position of the major papilla (inside, adjacent, or outside of the diverticula) in ERCP examination, which have become generally accepted for the classification of PAD [3]. The clinical importance of PAD originates from its association with pancreaticobiliary disease. Several studies have suggested that PAD is the reason for some clinical conditions, such as choledolithiasis and pancreatic disorders [1, 2]. However, the clinical characteristics associated with different types of PAD have not been well investigated. Therefore, we conducted this observational study to investigate the association of different types of PAD with occurrent and recurrent bile duct stones, with pancreatitis, and with the technical success of ERCP.

## 2. Methods 

### 2.1. Patients

The study included 850 consecutive patients who underwent their first ERCP during the period from August 2008 until December 2012. ERCP was performed when the imaging study and laboratory tests indicated that therapeutic management was needed or the diagnosis was uncertain.

Demographic characteristics, clinical information, imaging studies, and technical details and findings from an ERCP regarding those patients were entered into a database.

After completion of database entry for each patient with PAD, matched cases were selected into the non-PAD group (control group) that had corresponding parameters for age and gender. We adopted a 1 : 3 ratio of case: control proportion among the 850 consecutive patients who underwent ERCP during the study period. Of these, 161 patients (18.9%) had diverticula and the age- and sex-matched control group comprised 483 patients. This study was approved by the institutional review board of the hospital and informed consent was obtained from each patient.

### 2.2. Classification of PAD

A PAD was defined endoscopically as a depressed lesion of 5 mm or more with intact mucosa within a radius of 2.5 cm of the papilla [4]. PAD were classified as type 1, 2, or 3 according to the position of the major papilla from the endoscopic view [5]: type 1, the major papilla was located inside of the diverticula; type 2, the major papilla was located at the edge of the diverticula; type 3, the major papilla was located outside of the diverticula. The sizes of PAD were measured by using a Triple-Lumen Sphincterotome with a scale on the tip (Ultratome*™* XL Triple-Lumen Sphincterotome, model number M00535900, BOSTON SCIENTIFIC) during ERCP. The largest diameter of the PAD among length, breadth, and height was chosen as its representative.

### 2.3. Methods

The examinations were performed using a standard technique and duodenoscopes by a hepatobiliary surgeon. Successful cannulation was defined as free and deep instrumentation of the biliary tree and a cannulation attempt was defined as sustained contact with the cannulating device and the papilla for at least five seconds [6]. Post-ERCP complications include post-ERCP pancreatitis and gastrointestinal perforation.

### 2.4. Assessment of Recurrent CBD Stones

Patients with CBD stones who underwent the therapeutic ERCP among the 850 patients were followed up until the date of last follow-up as of January 2015. The recurrence of CBD stones was defined as the development of stones according to appropriate imaging studies not earlier than 6 months after the confirmation of complete removal of the CBD stones by ERCP. The exclusion criteria specified a recurrence of CBD stones within 6 months after ERCP. Recurrence-free survival was measured from the complete stones removal to occurrence of new onset, imaging-proven biliary stones requiring hospitalization for ERCP. Data on patients who were recurrence-free were censored on the date of last follow-up.

### 2.5. Statistical Analysis

For statistical analysis of the categorical data, the chi-square test or Fisher exact test was used. To evaluate the effect of the continuous variable, Student's *t*-test was used. Odds ratios and their 95% confidence intervals were calculated. For adjustment for possible confounders and effect modifiers, multivariate analyses were performed using logistic regression model. The actuarial probability curves for patients remaining free of recurrence of symptomatic CBD stones were constructed using the Kaplan-Meier analysis and compared with the log-rank test. All data analyses were performed using the SPSS statistical software program, version 19.0 (SPSS Inc., Chicago, IL, USA) for Windows and GraphPad Prism 5 (GraphPad Software Inc., San Diego, CA). *P* < 0.05 was considered as statistically significant.

## 3. Results 

### 3.1. Clinical Characteristics according to the Presence of PAD

There were 161 patients (18.9%) with 1 or more diverticula for whom sufficient data were available for this study. A single diverticula was evident in 80.7% of patients with PAD, 18.6% had 2 diverticula, and 0.7% had more than 2 diverticula. As shown in Table 1, the age and male-to-female ratio between the two study groups were balanced.

The incidences of biliary tract disorders in patients with PAD and controls are shown in Table 1. PAD was correlated with CBD stones (59.6% versus 35.0% in controls; *P* < 0.001) as well as with a higher previous cholecystectomy rate (39.1% versus 24.0% in controls; *P* < 0.001). However, there was no significant difference in the incidence of gall stones only (7.5% versus 9.5% in controls; *P* = 0.428) and gall stones with CBD stones (6.2% versus 11.4% in controls; *P* = 0.068) between the PAD group and the control group. Interestingly, compared to the control group, the detection rate of benign bile duct strictures (8.1% versus 14.7% in controls; *P* = 0.031) and periampullary carcinoma (6.8% versus 21.5% in controls; *P* < 0.001) was significantly lower in the PAD group.

In patients with PAD, acute pancreatitis, defined as pain and serum amylase elevation more than 3 times the normal value, was not found significantly more often than in control patients (31.7% versus 25.9%; *P* = 0.154). Chronic pancreatitis was found with equal frequency in both groups, 3.1% in PAD versus 1.9% (*P* = 0.355) in controls (Table 1).

Regarding the technical success of ERCP, there were no significant differences between the PAD group and controls in terms of successful duct cannulation (95.0% versus 91.9% in controls; *P* = 0.190). Severe post-ERCP pancreatitis, defined as abdominal pain and serum amylase elevation of 3 times the normal value, was observed in 16.1% of PAD group patients and 12.6% of controls (*P* = 0.258). Retroperitoneal perforation was rarely seen and no difference was detected in the perforation rate between two groups (1.2% versus 2.5% in controls; *P* = 0.535) (Table 1).

The abovementioned univariate *P* values have to be regarded as descriptive. For adjustment for possible confounders and effect modifiers, a multivariate logistic regression model was used with the independent variables which are confirmed to be statistically significant by univariate analysis (Table 1). If those confirmatory multivariate *P* values are considered, CBD stones only (*P* = 0.008) and periampullary malignancy (*P* = 0.004) remain significant (Table 1).

### 3.2. Clinical Characteristics according to the PAD Subtypes

The relative frequency of PAD was further stratified according to the subtype: 9.9% of PAD was diagnosed as type 1, 28.0% as type 2, and 62.1% as type 3. The existence of PAD subtypes was correlated with differences in clinical characteristics (Table 2).

The PAD size (mean ± SD) in patients with type 1 PAD was 18.9 ± 9.2 mm, which was significantly larger than that in patients with type 2 PAD (12.1 ± 4.7 mm, *P* = 0.003) or type 3 PAD (10.6 ± 8.6 mm, *P* < 0.001). Similarly, the occurrence of acute pancreatitis was more frequent (62.5%) in patients with type 1 PAD, and it was approximately 2 times higher than patients with type 2 PAD (28.9%, *P* = 0.017) or type 3 PAD (28.0%, *P* = 0.006). In addition, CBD stones alone showed higher tendency in type 1 PAD than in type 2 or type 3 PAD although it did not reach a statistical significance. Moreover, there were no significantly different characteristics between patients with type 2 and type 3 PAD.

### 3.3. Risk Factors for the Recurrence of Symptomatic CBD Stones

330 patients were diagnosed to have symptomatic CBD stones in this study. In order to study the risk factors of recurrence of symptomatic CBD stones after therapeutic ERCP, a total of 301 patients were finally enrolled (29 patients were excluded due to the follow-up loss or recurrence of CBD stones within 6 months after ERCP). The median follow-up was 40 months (6–76 months). The recurrence of CBD stones occurred in 32 patients (10.6%) during the follow-up period, and the median time until the first recurrence was 36 months (6–60 months).

After the univariate analysis and the multivariate logistic regression analysis of the potential risk factors of the recurrence of symptomatic CBD stones after therapeutic ERCP, we found that PAD (odds ratio [OR] = 2.968, [95% CI, 1.394–6.321], *P* = 0.005) and prior cholecystectomy (odds ratio [OR] = 3.106, [95% CI, 1.287–7.496], *P* = 0.012) were the two independent risk factors (Table 3).

**Figure 1:**
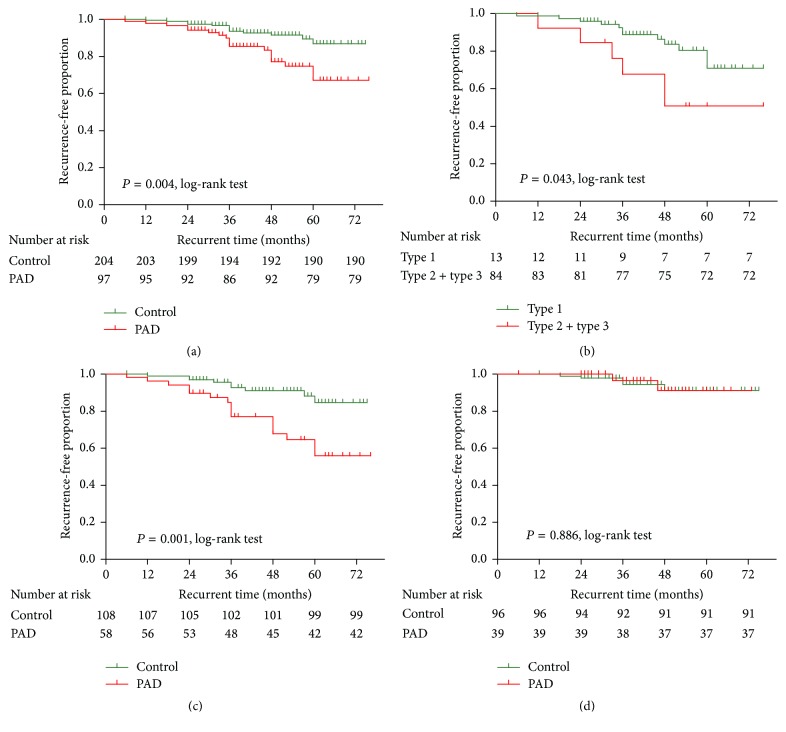
The actuarial probability of patients remaining free of recurrence of symptomatic CBD stones during the follow-up after therapeutic ERCP. (a) The patients with PAD versus those without PAD; (b) the patients with type 1 PAD versus those with type 2 or type 3 PAD; (c) recurrence-free probability between the two groups who underwent previous cholecystectomy; (d) recurrence-free probability between the two groups with gall bladder in situ.

The actuarial probability of patients remaining free of recurrence of symptomatic CBD stones during the follow-up after therapeutic ERCP with PAD was significantly lower than that for the patients without PAD (81.4% versus 93.1%, resp.; *P* = 0.004, log-rank test) (Figure 1(a)). Subgroup analysis showed that the patients with type 1 PAD had significantly lower rates of being free of recurrence of CBD stones during the follow-up than did the patients who had type 2 or type 3 PAD (53.8% versus 85.7%, resp.; *P* = 0.043, log-rank test) (Figure 1(b)). Since PAD and prior cholecystectomy were two independent risk factors for recurrence of symptomatic CBD stones after therapeutic ERCP, another subgroup analysis was performed to determine if there was a differential recurrence-free probability between patients with or without PAD who underwent previous cholecystectomy. The PAD patients who underwent previous cholecystectomy had a significantly lower rate of being free of recurrence of CBD stones than the patients without PAD (42/58, 72.4% versus 99/108, 91.7%, resp.; *P* = 0.001, log-rank test) (Figure 1(c)), whereas in patients with gall bladder in situ, PAD did not have a promoting effect on recurrence of CBD stones as the recurrence-free rate was similar between the patients with or without PAD (37/39, 94.9% versus 91/96, 94.8%, resp.; *P* = 0.886, log-rank test) (Figure 1(d)). However, due to the small number of recurrences in patients with gall bladder in situ, the statistical power was limited.

## 4. Discussion

In this study, we sought to demonstrate the association of different types of PAD with occurrent and recurrent bile duct stones, with pancreatitis, and with the technical success of ERCP.

PAD, not uncommon findings during ERCP, has been reported to be associated with biliary diseases [2]. Since the incidence of both PAD and bile duct stones increases with age [7, 8], our study adjusted this confounding variable and found that the prevalence of PAD was increased in patients with CBD stones but not in those with gallbladder stones alone, which confirms the findings from other reports that PAD is associated with bile duct stones [9–11]. Furthermore, type 1 PAD, in which the major papilla is located within the diverticula, is considered to carry a theoretically greater risk of biliary stones formation [5]. Consistent with this speculation, we noted that common bile duct stones were relatively more common in type 1 PAD patients (81.7%) when compared to patients with type 2 and type 3 PAD (57.8%, 57.0%, resp.) although it did not reach to a statistical difference. As such, these results strongly suggest a causal relationship between PAD and biliary stones formation. The pathological mechanism of this association is explained by several hypotheses. The mechanical pressure of the diverticula to the distal end of the biliary tract is commonly discussed. The larger the PAD and the closer it is to a papilla, the more it may disturb the bile flow [12]. Another hypothesis is related to the dysfunction of the sphincter of Oddi (SO), which is believed to be caused by the accumulation of food in the diverticula, compressing the end of the bile duct as well as SO and leading to stricture of the sphincter. The dysfunction of the SO leads to the reflux of gastrointestinal juice into the bile duct, bacterial infection of the bile duct, and formation of the pigment bile duct stones [1, 3, 13, 14].

In addition, we noted that the presence of PAD is negatively associated with prevalence of periampullary carcinoma which to our knowledge has not been reported before. However, there were some studies trying to investigate the relationship between diverticulosis, diverticulitis, polyps, advanced neoplastic lesions (ANL), and colorectal carcinoma (CRC). One study from Korea found an increased risk of CRC in both patients with left- or right-sided diverticulosis without prior polypectomy or surgery in the affected area [15]. One of the possible explanations for the association between diverticular disease and colorectal cancer is that the presence of inflammation process increases the risk of malignant transformation. In contrast, three studies [16–18] showed no relationship between diverticulosis and CRC, one of them being a longitudinal study [16]. Moreover, another three studies [19–21] found less CRC in patients with diverticular disease, which speak in favor of what we observed that periampullary malignancy is less common in PAD patients. One possible explanation is that an altered matrix composition predisposes to the development of colon cancer in the colonic tissue architecture of cancer patients but not in patients with diverticular disease [22]. According to these literature reviews, we considered one hypothesis to explain the negative correlation between periampullary malignancy and PAD. That is, there may be a more protumorigenic matrix microenvironment in the periampullary tissue architecture in the patients without PAD compared to those with PAD, which is in accordance with no predisposition for cancer in diverticular disease in these patients. Starting from this point, our future work is to investigate the detailed microenvironment composition of periampullary tissue in patients with periampullary malignancy or patients with different types of PAD by using tissue and gene microarray analyses. These approaches would be a powerful tool in grouping cancer patients into classes with clinical and therapeutic relevance. Clearly, the longitudinal study following cohorts of patients with diverticulosis or diverticulitis instead of cross-sectional study is the best way to analyze the causality between diverticulosis and periampullary malignancy. Therefore, we will also start a longitudinal study in the near future following cohorts of patients with PAD and try to clarify this causality.

It has been a matter of dispute whether or not pancreatitis is induced by PAD per se. Some investigators have suggested that pancreatitis is not associated with PAD [9, 23]. Others reported that patients with PAD have a higher rate of acute pancreatitis [24, 25]. Our study did not find a significant higher rate of pancreatitis in PAD patients than in patients without PAD. However, we found that the type 1 PAD patients had a bigger PAD size and a higher frequency of acute pancreatitis than the patients with type 2 or type 3 PAD. From this observation we might hypothesize that the distension of diverticula with specific location (papilla located inside of the diverticula) may cause compression of the pancreatic duct and result in pancreatitis. As described above, PAD predispose the patient to common bile duct stones; it is difficult to tell whether pancreatitis is from biliary origin or by the diverticula themselves. But at least, the presence of PAD should be taken into account, mainly in elderly patients, before defining a pancreatitis as idiopathic.

PAD is thought to be an impediment to ERCP procedures. Although successful cannulation in patients with PAD varies from 61% to 95.4%, this was found to be significantly lower compared with patients without PAD in some studies [24, 26, 27]; however, some other papers showed that the successful cannulation rate and morbidity and mortality rates after ERCP were almost the same between patients with and without PAD [4, 9, 28, 29]. The various techniques for cannulation, the experience of the operators, the different patient characteristics, and the lack of adjustment for those variables between the exposed and control groups can all be responsible for explaining the lack of consistency in results so far. Our study has the advantage of including a concrete sample of Chinese patients treated by the same experienced surgeon in a university hospital. We found no difference in successful cannulation between patients with and without PAD, irrespective of the location of the papilla.

In clinical practice, a considerate number of patients visit the hospital for management of the recurrence of symptomatic CBD stones. In this situation, identifying the risk factors for the development of recurrent CBD stones is needed. In this study, the independent risk factors for the recurrence of symptomatic CBD stones were PAD and prior cholecystectomy. PAD has been advocated as a factor for recurrence of CBD stones in several previous studies [30–32], yet this is still controversial. Ando et al. [14] did not regard the periampullary diverticula as a risk factor for recurrent bile duct stones after endoscopic papillotomy. Kim et al. [33] addressed that periampullary diverticula is associated with patients with primary common bile duct stones, but not with the secondary ones. However, in our study, the presence of PAD was the independent risk factor of the recurrence of symptomatic CBD stones after therapeutic ERCP. More specifically, type 1 PAD, with the papilla located within the diverticula, was correlated with a shorter recurrence time of symptomatic CBD stones. This is consistent with the study of Kim et al. [5] and the study of Baek et al. [34] which both suggested that type 1 PAD was related to recurrence of CBD stones. The factor of prior cholecystectomy was considered as another independent risk factor for recurrence of CBD stones in our study. In subgroup analysis we found that, in the patients with an intact gall bladder, PAD did not increase the CBD stones recurrence rate. It is probable that gall bladder motility is related to the low recurrence rate of CBD stones. Several authors proved that significant improvement in gall bladder motility was achieved after therapeutic ERCP [35, 36]. It is also noted that bile stasis is an important factor in the pathogenesis of bile duct stone formation. Frossard et al. [37] evaluated 92 patients with CBD stones and reported that the presence of the gall bladder was significantly associated with spontaneous bile duct stone passage. These positive roles of gall bladder may neutralize the ill effects of PAD in the recurrence of symptomatic CBD stones. However, in patients with prior cholecystectomy, the relative risk of the PAD group was significantly higher than that of the group without PAD (risk ratio [RR] = 4.034, [95% CI, 1.742–9.346], *P* = 0.001). It is probable that cholecystectomy can result in some secondary changes like the dysfunction of the sphincter of Oddi, common bile duct dilatation, long cystic duct stump, and bile duct angulation which were very important factors in the pathogenesis of bile duct stone formation [38–40]. In addition, the slow biliary emptying and bile stasis in patients with PAD [30] may have a synergetic effect with the secondary changes induced by cholecystectomy in forming the recurrence of CBD stones. Therefore, careful periodic surveillance of blood tests, ultrasonography, and/or magnetic resonance cholangiography may be recommended for CBD patients who present with PAD with prior cholecystectomy after therapeutic ERCP.

In conclusion, this study demonstrates that PAD, especially type 1 PAD, is associated with an increased occurrence and recurrence of CBD stones. Acute pancreatitis is more frequent in patients with type 1 PAD than patients with type 2 or type 3 PAD. PAD during an ERCP should not be considered an obstacle to a successful cannulation.

## Figures and Tables

**Table 1:** Comparison of cholangiopancreatic disorders and technical success of ERCP according to the presence of PAD.

	PAD (*n* = 161)	Control (*n* = 483)	*P*_*u*_^*∗*^	*P*_*m*_^†^	Odds ratio [95% CI]
Median age (yr) (range)	62 (23–90)	61 (26–87)	0.542		
Gender: *n* (%)					
Male	83 (51.6)	249 (51.6)	1.000		
Female	78 (48.4)	234 (48.4)			
Biliary disorders: *n* (%)					
CBD and gall stones	10 (6.2)	55 (11.4)	0.068		
CBD stones only	96 (59.6)	169 (35.0)	<0.001	0.008	2.09 [1.213–3.602]
Gall stones only	12 (7.5)	46 (9.5)	0.428		
Benign bile duct strictures	13 (8.1)	71 (14.7)	0.031	0.154	0.61 [0.311–1.203]
Periampullary carcinoma	11 (6.8)	104 (21.5)	<0.001	0.004	0.35 [0.174–0.710]
Previous cholecystectomy	63 (39.1)	116 (24.0)	<0.001	0.712	1.10 [0.653–1.867]
Pancreatic disorders: *n* (%)					
Acute pancreatitis	51 (31.7)	125 (25.9)	0.153		
Chronic pancreatitis	5 (3.1)	9 (1.9)	0.349		
Successful cannulation: *n* (%)	153 (95.0)	444 (91.9)	0.190		
Complications: *n* (%)					
Post-ERCP pancreatitis	26 (16.1)	61 (12.6)	0.258		
Perforation	2 (1.2)	12 (2.5)	0.533		

^**∗**^Student's *t*-test for continuous variables and Pearson's chi-square test for categorical variables.

^†^The multivariate logistic regression model included variables which are confirmed to be statistically significant by univariate analysis as independent variables and PAD as dependent variable.

CBD: common bile duct; ERCP: endoscopic retrograde cholangiopancreatography.

**Table 2:** Cholangiopancreatic disorders and technical success of ERCP in different PAD subtypes.

	Type 1 (*n* = 16)	Type 2 (*n* = 45)	Type 3 (*n* = 100)	*P* value^*∗*^
Median age (yr) (range)	65 (51–78)	66 (24–90)	58 (23–88)	0.134
Gender: *n* (%)				
Male	9 (56.3)	28 (62.2)	46 (46.0)	0.180
Female	7 (43.8)	17 (37.8)	54 (54.0)	
PAD size (mean ± SD, mm)	18.9 ± 9.2	12.1 ± 4.7	10.6 ± 8.6	0.001
Biliary disorders: *n* (%)				
CBD stones and gall stones	2 (12.5)	3 (6.7)	5 (5.0)	0.508
CBD stones only	13 (81.3)	26 (57.8)	57 (57.0)	0.177
Gall stones only	1 (6.3)	3 (6.7)	8 (8.0)	0.943
Benign bile duct strictures	0 (0.0)	4 (8.9)	9 (9.0)	0.458
Periampullary carcinoma	0 (0.0)	2 (4.4)	9 (9.0)	0.314
Previous cholecystectomy	9 (56.3)	15 (33.3)	39 (39.0)	0.272
Pancreatic disorders: *n* (%)				
Acute pancreatitis	10 (62.5)	13 (28.9)	28 (28.0)	0.020
Chronic pancreatitis	1 (6.3)	2 (4.4)	2 (2.0)	0.549
Successful cannulation: *n* (%)	15 (93.8)	44 (97.8)	94 (94.0)	0.607
Complications: *n* (%)				
Post-ERCP pancreatitis	2 (12.5)	8 (17.8)	16 (16.0)	0.884
Perforation	0 (0.0)	1 (2.2)	1 (1.0)	0.740

^*∗*^One-way analysis of variance for continuous variables and Pearson's chi-square test or Fisher exact test for categorical variables.

CBD: common bile duct; ERCP: endoscopic retrograde cholangiopancreatography.

**Table 3:** Univariate analysis of the risk factors for recurrence of symptomatic CBD stones.

	Recurrence group (*n* = 32)	Nonrecurrence group (*n* = 269)	*P* value^*∗*^
Median age (yr) (range)	62.5 (44–78)	61 (27–90)	0.360
Gender: *n* (%)			
Male	18 (56.3)	131 (48.7)	0.419
Female	14 (43.8)	138 (51.3)	
CBD diameter (mean ± SD, mm)	14.5 ± 4.6	14.3 ± 8.3	0.957
CBD stone size (mean ± SD, mm)	11.5 ± 6.4	11.5 ± 7.1	0.984
CBD stone number: *n* (%)			
1	8 (25.0)	93 (34.6)	0.278
≥2	24 (75.0)	176 (65.4)	
PAD: *n* (%)	18 (56.3)	79 (29.4)	0.002
Type 1	6 (18.8)	7 (2.6)	
Type 2 + type 3	12 (37.5)	72 (26.8)	
Prior cholecystectomy: *n* (%)	25 (78.1)	141 (52.4)	0.006
ERCP attempt: *n* (%)			
1	26 (81.2)	239 (88.8)	0.211
≥2	6 (18.8)	30 (11.2)	
Lithotripsy: *n* (%)	6 (18.8)	37 (13.8)	0.445
EST: *n* (%)	22 (68.8)	214 (79.6)	0.160
EPBD: *n* (%)	24 (75.0)	221 (82.2)	0.325
Successful cannulation: *n* (%)	31 (88.1)	256 (93.4)	0.665

^**∗**^Student's *t*-test for continuous variables and Pearson's chi-square test for categorical variables.

CBD: common bile duct; EST: endoscopic sphincterotomy; EPBD: endoscopic papillary balloon dilation.
